# Characteristics of Meat from Farmed Sika Deer (*Cervus nippon*) and the Effects of Age and Sex on Meat Quality

**DOI:** 10.3390/foods13233978

**Published:** 2024-12-09

**Authors:** Zhangrong Peng, Hui Zhao, Jing Luo, Haoran Sun, Qingkui Jiang, Tietao Zhang

**Affiliations:** 1 Institute of Special Animal and Plant Sciences, Chinese Academy of Agricultural Sciences, Changchun 130117, China; pzr13541494656@163.com (Z.P.); baobeihuihui815@163.com (H.Z.); luojing01@caas.cn (J.L.); solomoncat@163.com (H.S.); 2 Public Health Research Institute, New Jersey Medical School, Rutgers Biomedical and Health Sciences, Rutgers, The State University of New Jersey, Newark, NJ 07103, USA

**Keywords:** Sika deer, nutritional quality, physical characteristics, age, sex

## Abstract

This study assessed the meat quality of Sika deer (*Cervus nippon*) from various age and sex groups using the longissimus dorsi (LD) muscle. Samples from different age groups (2, 3, and 4 years old) were analyzed for various parameters. The results show that, with increasing age, there is a decrease in moisture and drip loss (*p* < 0.05), alongside increases in ash, protein, fat, and cooking loss (*p* < 0.05). Female deer showed an increase in tenderness (*p* < 0.05), whereas males generally had a higher amino acid content (*p* < 0.05). Interestingly, 2-year-old female Sika deer had more saturated fatty acids (*p* < 0.05), while 3- and 4-year-old females had more unsaturated fatty acids compared to age-matched males (*p* < 0.05). Notably, 3-year-olds had higher levels of monounsaturated and polyunsaturated fatty acids in both the male and female groups (*p* < 0.05). Overall, this study provides the first comprehensive evidence that Sika deer meat is a nutritious source of lean protein. Notably, meat from 3-year-old Sika deer, regardless of sex, contains higher nutrient levels and is more tender compared to meat from deer of other ages. Additionally, meat from females tends to be more tender than that from males.

## 1. Introduction

Deer, a diverse group of herbivorous mammals, are widely distributed across the globe, with species such as roe deer (*Capreolus capreolus*), red deer (*Cervus elaphus*), fallow deer (*Dama dama*), and Sika deer (*Cervus nippon*) being commonly hunted or farmed for various products [1,2,3,4,5]. Among these, Sika deer farming is notably prominent in China, where approximately 700,000 Sika deer are reared annually, making the country a leader in Sika deer production [1,6,7]. While deer velvet is the most popular product for medicinal purposes in Asia, venison has gained global recognition for its reputation as a healthy, low-fat, and organic meat option [8], which has contributed to a continuous increase in the demand for deer meat in both Asia and the international market [4].

Historically, venison is produced from wild deer, but rising demand has led an increase in farmed deer production [9], allowing venison to be more accessible to average consumers. Compared to wild deer, farmed deer receive a stable diet, resulting in meat with more consistent flavor and quality [10]. Conversely, meat from wild deer, including Sika deer, may exhibit low quality due to insufficient and unbalanced food sources, environment, and stress levels [11]. Previous research has extensively examined the nutritional composition and physicochemical attributes of venison from species like roe deer, red deer, and fallow deer [9,10,12,13,14,15,16,17]. However, much of these work has focused on wild deer, with limited attention given to the chemical composition of farmed deer meat, particularly that of farmed Chinese Sika deer.

This study aims to addresses this knowledge gap by evaluating the physicochemical properties and nutrient composition of meat from farmed Chinese Sika deer. Specifically, we assess the moisture, color, pH, shear force, cooking loss, amino acid profile, and fatty acid composition, and investigate how these quality attributes vary with age and sex. The findings from this research will provide industry professionals and consumers with a deeper understanding of Sika deer meat quality and offer insights for optimizing venison production and processing techniques.

## 2. Materials and Methods

### 2.1. Experimental Design and Animals

Male and female Sika deer, aged 1, 2, and 3 years, were selected from the Dong’ao Deer Experimental Base in Shuangyang City, Jilin Province, China (43°31′21.9″ N, 125°39′31.7″ E). Sika deer of these ages were selected for this study because they reach sexual maturity around one year of age, at which point their body growth rate slows, but muscle development accelerates [18]. This period of age represents a key phase for muscle growth and quality development, making these deer particularly suitable for investigating meat characteristics. The deer were assigned to nine groups: 2-year-old male (m2), 3-year-old male (m3), 4-year-old male (m4), 2-year-old female (f2), 3-year-old female (f3), and 4-year-old female (f4), with 3 bodyweight-matched animals per group. Each group was housed in individual pens and fed the experimental diet (Table S1) twice daily at 08:00 and 16:00 (Beijing time), with ad libitum access to feed and water. Regular veterinary examinations ensured the health status of all animals. After a one-year experimental period, the animals were slaughtered on farm, and then the longissimus dorsi (LD) muscle was collected after skinning and evisceration for further analysis.

### 2.2. Preparation of Meat Samples

In this study, all meat samples from Sika deer were collected from February 2023 to March 2023 in Shuangyang, the “deer town” of Sika deer in China. The requirements of the test animals were categorized into three age groups, 2, 3, and 4 years old, of which three males and three females were in each age group, for a total of 18 Sika deer. Immediately after slaughter, approximately 900 g samples were collected from each animal and brought back to the laboratory in a self-made sampling kit with ice packs; a portion of the venison was taken to carry out the meat physicochemical tests immediately, and the rest of the meat was kept in −20 °C for further analysis.

For nutrient content determination, the frozen samples were thawed to remove the fascia, and then ground using a meat grinder (Ultron Technology, Foshan, China). Ten percent of the minced meat was allocated for determination of moisture content. The remaining portion was prepared for nutrient analysis. To ensure accuracy and reliability of the measurements, samples were baked flat in a GZX-9240MBE drying oven (Boxun, Shanghai, China) at 50 °C for 48 h, following AOAC Method 984.13 [19]. This process maximized moisture removal and preserved the sample optimally. After drying, the samples were crushed into a fine powder and sieved through a 60-mesh sieve to obtain uniform particles for further analyses.

### 2.3. Chemical Characteristic Analysis

The nutritional quality of the meat was analyzed according to the methods of AOAC [19]. Briefly, crude protein content was determined by NDA 701 Dumas Nitrogen Analyzer (VELP Scientifica, Usmate, Italy), fat content by a SEF-06C Soxhlet Fat Analyzer (Wincom, Changsha, China), and ash content by SX2-6-12TP muffle furnace (Zhenhua Instruments, Taizhou, China) (methods 984.13, 920.39, and 967.05, respectively). Moisture content was determined by the direct drying method as in [19].

### 2.4. Amino Acid Profile Analysis

Amino acid content was determined by an L-8900 automatic amino acid analyzer (Hitachi, Tokyo, Japan) using the AOAC method 994.12 [19]. Weigh 50.0 mg ± 1.0 mg of air-dried venison sample powder in the amino acid hydrolysis tube; then, add 20 mL of hydrochloric acid solution with a concentration of 6 mol/L, tighten the screw cap, and put it in the drying oven at 110 °C for hydrolysis for 22 h. After the end of hydrolysis and cooled down to room temperature, 800 μL of hydrolysis solution was sucked up and put into a vacuum-drying oven, evaporated under reduced pressure at 70 °C, and then 2 mL of hydrochloric acid solution with a concentration of 0.02 mol/L of hydrochloric acid solution was added, filtered through a 0.22 μm aqueous needle filter to the sample bottle, and then analyzed by the automatic amino acid analyzer. For the separation column, we have the following properties: citric acid-sodium citrate buffer at a flow rate of 0.4 mL/min, column temperature of 50 °C, and column pressure of 9.0 MPa; for the chromatographic column, we have the following properties: Hitachi 2622C sulfonic acid cationic resin separation column (4.6 mm × 60 mm, 3 μm) (Hitachi, Tokyo, Japan); and, for the gradient elution, we have the following properties: cycle time of 53 min. The reaction column was operated at a flow rate of 0.35 mL/min for ninhydrin, with a column temperature of 135 °C and a column pressure of 1.1 MPa. The ultraviolet detector was used to detect proline at a wavelength of 570 nm for 10 min and the other 16 amino acids at 440 nm for 32 min.

### 2.5. Fatty Acid Profile Analysis

Fatty acid content was determined as previously described [20]. Briefly, the following conditions were adapted: capillary column: DB-23 (60 mm × 0.25 mm × 0.25 μm) (Agilent, Folsom, CA, USA); the carrier gas was high-purity helium at a flow rate of 1 mL/min; the inlet temperature was 230 °C, and the injection volume was 1 μL; and the programmed heating conditions were as follows: the initial temperature was 60 °C, held for 1 min, then the temperature was increased to 180 °C at 6 °C/min and increased to 180–220 °C at 2.5 °C/min and held for 5 min. The temperature was increased to 180–220 °C at 2.5 °C/min and maintained for 5 min.

The following parameters were calculated by the content of individual fatty acids (FA): SFAs: saturated fatty acids; UFAs: unsaturated fatty acids; PUFAs: polyunsaturated fatty acids; MUFAs: monounsaturated fatty acids; n-6 PUFA: polyunsaturated fatty acid; and AI: atherogenic index, calculated as: AI = [C12:0 + 4 × C14:0 + C16:0]/[MUFA + PUFA].

### 2.6. Physical Traits

Meat color was determined using a Minolta CR-400 Color Chroma Meter (Konica Minolta Sensing Americas, Ramsey, NJ, USA), according to the manufacturer’s instruction. Briefly, the illuminant setting was set to D65, representing daylight conditions at 6500 K, with a 10° observer angle. For each sample, measurements were taken from three unique locations (avoiding edges) of uniform thickness were taken and then averaged. The color parameters were recorded as L* (brightness), a* (red), and b* (yellow) [21].

pH was determined using a PHSJ-3F pH meter (Yantai Stark Instrument Co., Yantai, China). Briefly, three representative sections were collected from the interior of each meat sample. Each section was homogenized and measured separately [3], and the pH values were averaged to obtain a final reading for each sample.

Cooking loss was determined as previously reported in [22]. Briefly, samples (30 g ± 0.1 g) were weighed before cooking, the cooking bag was placed in a Dk-420 water bath (Wincom, Changsha, China) at 80 °C, the center temperature of the meat samples was determined by a 53 II thermocouple thermometer (Fluke, Everett, WA, USA) to reach 70 °C when the meat samples were taken out, and the weight of the meat samples was determined after cooling down to room temperature (about half an hour) by wiping off the surface water. Cooking loss (%) = ((weight of raw meat-weight of cooked meat)/weight of raw meat) × 100.

Intact meat samples used for cooking loss analysis was used for tenderness analysis using a Universal Testing Machine 5542 (Intron, Norwood, MA, USA) attached with a Warner-Bratzler ‘V’ Slot Blade (G-R Manufacturing, Manhattan, KS, USA) following manufacturer’s instruction. Three to five measurements from different areas within each sample were taken and averaged to obtain a representative tenderness value [23].

The 24 h and 48 h drip loss were determined as described in [22]. Briefly, fresh meat samples (10 g ± 0.1 g) were weighed and suspended using an “S” hook attached to one end. Each sample was carefully placed in a plastic bag, ensuring that the meat did not come into contact with the bag. The samples were suspended at 4 °C with the mouth of the bag tightly secured. After 24 h, the samples were removed, weighed, and then suspended again under the same conditions for an additional 24 h (48 h total). Drip loss at each timepoint was calculated as follows: Drip loss (%) = (initial weight of meat − weight of meat after hanging)/initial weight of meat × 100.

### 2.7. Statistical Analysis

The experimental data were analyzed by two-way ANOVA using SPSS 26.0 (IBM, Armonk, NY, USA) to compare the effects of age and sex on different detection indices and the interaction between age and sex, respectively. Tukey’s method was used to test the differences between each pair of groups. Pearson correlation analysis and principal component analysis were plotted using R software (version 4.3.0). All experimental data were expressed as mean ± standard deviation, and all tests were conducted in triplicate. Significance was considered at the level *p* < 0.05, with *p* < 0.01 as a highly significant difference.

## 3. Result

### 3.1. Chemical Characteristics of Sika Deer Meat

**Figure 1:**
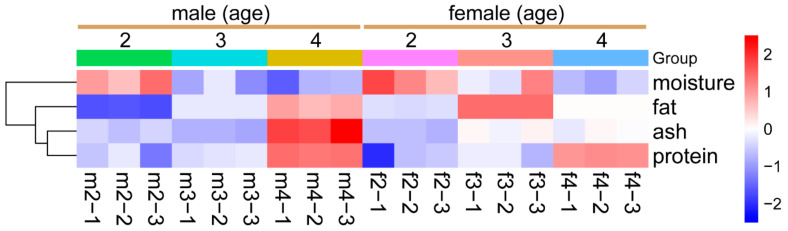
**Dynamic changes in chemical characteristics of Sika deer LD.** Heatmap showing the relative values of chemical characteristics in the LD muscle of Sika deer, categorized by different sex and age groups. Values are color-coded to indicate an increase (red) or decrease (blue) relative to the corresponding mean value of each parameter across all groups. The color intensity of the cells corresponds with the magnitude of change, with darker shades representing greater deviations from the mean, as indicated by the color scale.

Table 1 presents the effect of sex and age on the chemical composition of Sika deer meat. Our findings show that Sika deer LD has a protein and fat content of approximately 20–23% and 0.5–3% of fresh matter, respectively. While moisture decreased with age (*p* < 0.05), there was a general increase in protein, fat, and ash contents in both male and female Sika deer (*p* < 0.05) (Table 1, Figure 1). Females had a high content of fat and ash at 3 years of age compared to males (*p* < 0.05), but this trend reversed at 4 years of age (*p* < 0.05) (Table 1, Figure 1).

### 3.2. Physical Characteristics of Sika Deer Meat

**Figure 2:**
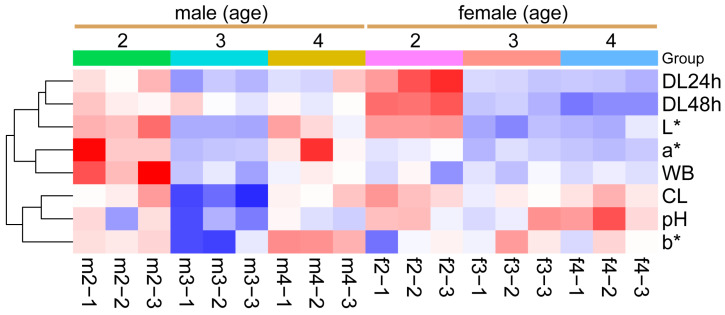
**Dynamic changes in physical characteristics of Sika deer LD.** Heatmap showing the relative values of physical characteristics in the LD muscle of Sika deer, categorized by different sex and age groups. Values are color-coded to indicate an increase (red) or decrease (blue) relative to the corresponding mean value of each parameter across all groups. The color intensity of the cells corresponds the magnitude of change, with darker shades representing greater deviations from the mean, as indicated by the color scale. WB: shear force; DL: drip loss; CL: cooking loss; L* = brightness; a* = redness; b* = yellowness. m2: 2-year-old male deer; m3: 3-year-old male deer; m4: 4-year-old male deer; f2: 2-year-old female deer; f3: 3-year-old female deer; f4: 4-year-old female deer.

Table 2 shows the physical characteristics of Sika deer LD. Shear force, cooking loss, and pH differed significantly between sexes (*p* < 0.01), with a lower shear force, higher cooking loss, and higher pH observed in female deer (*p* < 0.05). Drip loss decreased with age at both 24 h and 48 h in both genders (*p* < 0.05). While brightness (L*) decreased with age (*p* < 0.05), the redness (a*) and yellowness (b*) remained stable in female deer across all ages. However, in males, brightness, redness, and yellowness were the lowest at 3 years of age (*p* < 0.05) (Table 2, Figure 2).

### 3.3. Amino Acid Profile of Sika Deer Meat

**Figure 3:**
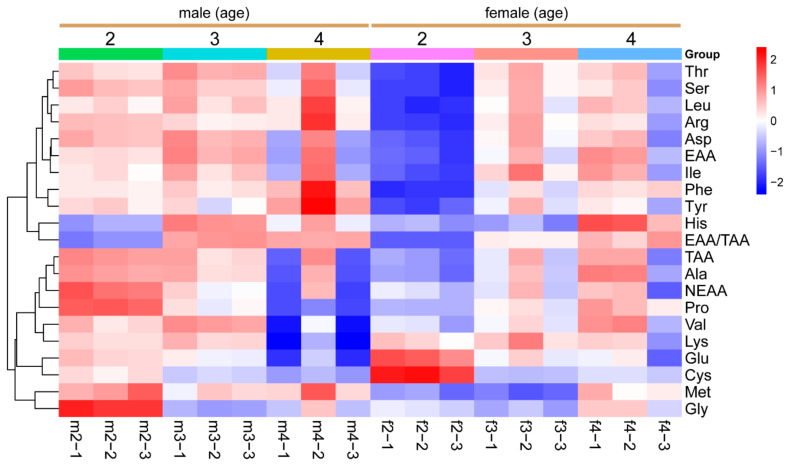
**Dynamic changes in amino acids in Sika deer LD.** Heatmap showing the relative values of amino acids in the LD muscle of Sika deer, categorized by different sex and age groups. Values are color-coded to indicate an increase (red) or decrease (blue) relative to the corresponding mean value of each parameter across all groups. The color intensity of the cells corresponds the magnitude of change, with darker shades representing greater deviations from the mean, as indicated by the color scale. TAA: total amino acid; EAA: essential amino acid; NEAA: non-essential amino acid; EAA/TAA: essential amino acid/total amino acid. m2: 2-year-old male deer; m3: 3-year-old male deer; m4: 4-year-old male deer; f2: 2-year-old female deer; f3: 3-year-old female deer; f4: 4-year-old female deer.

Table 3 presents the amino acid composition in the LD muscle of Sika deer across different sex and age groups. The two most abundant essential amino acids were lysine and leucine (6.18~7.50% and 5.30~6.73%, respectively), and the most abundant non-essential amino acids in Sika deer LD were glutamic acid and aspartic acid (10.90~13.89% and 6.12~7.26%, respectively). Age significantly influences the amino acid composition, but this influence varies between sexes. In males, younger deer showed higher levels of non-essential amino acids (NEAA) (*p* < 0.05), while, in females, older deer showed higher levels of essential amino acids (*p* < 0.05) (Table 3, Figure 3). Threonine, methionine, leucine, phenylalanine, and lysine are among the significantly affected amino acids by both age and gender (*p* < 0.05).

### 3.4. Fatty Acid Profile of Sika Deer Meat

**Figure 4:**
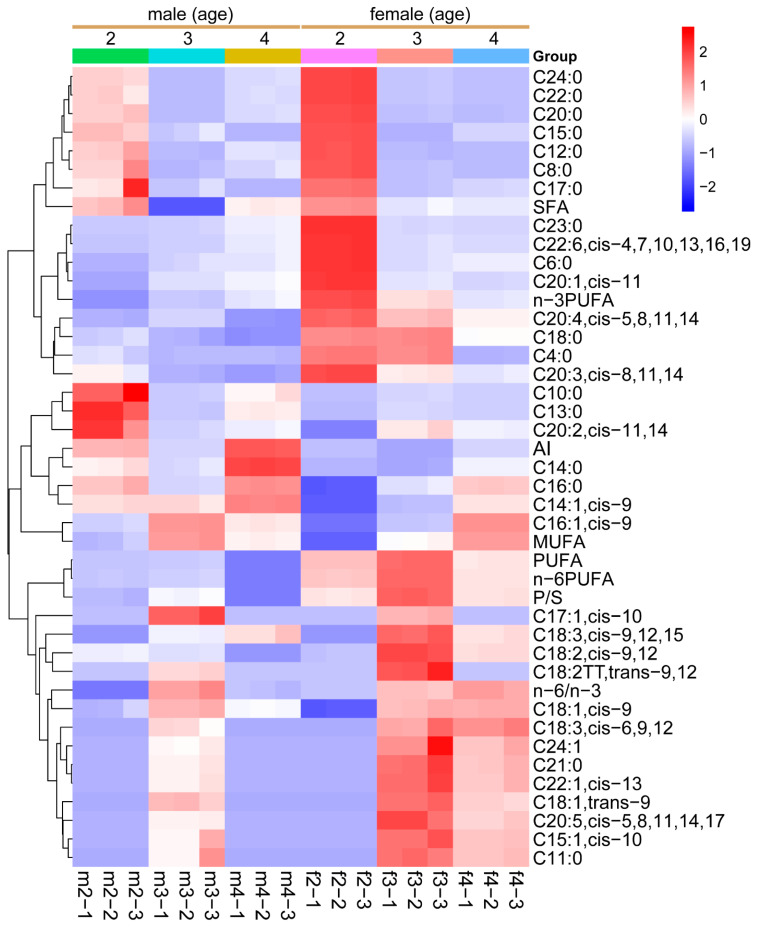
**Dynamic changes in fatty acids in Sika deer LD.** Heatmap showing the relative values of fatty acids in the LD muscle of Sika deer, categorized by different sex and age groups. Values are color-coded to indicate an increase (red) or decrease (blue) relative to the corresponding mean value of each parameter across all groups. The color intensity of the cells corresponds the magnitude of change, with darker shades representing greater deviations from the mean, as indicated by the color scale. SFA: saturated fatty acid; MUFA: monounsaturated fatty acid; PUFA: polyunsaturated fatty acid; n-6/n-3: n-6PUFA/n-3PUFA; P/S: PUFA/SFA; AI: atherogenic index. m2: 2-year-old male deer; m3: 3-year-old male deer; m4: 4-year-old male deer; f2: 2-year-old female deer; f3: 3-year-old female deer; f4: 4-year-old female deer.

Table 4 shows the fatty acid profile of Sika deer LD and the influence of sex and age on it. A total of 35 fatty acids were detected. Our findings indicate that Sika deer meat is rich in C16:0, C16:1, C18:0, C18:1n9c, and C18:2n6c. Saturated fatty acid (SFA) content, such as C4:0, C10:0, and C18:0, is generally higher in females than in males across most ages (*p* < 0.05). Females generally show higher monounsaturated fatty acid (MUFA) levels, particularly at older ages (*p* < 0.05). In contrast, males show lower levels of MUFAs at younger ages but these levels increase as they age (*p* < 0.05). Additionally, females have considerably higher levels of polyunsaturated fatty acids (PUFAs), including n-3 and n-6 PUFAs, C20:4, and C22:6, especially at older ages (*p* < 0.05) (Table 4; Figure 4).

### 3.5. Correlation Among Meat Quality Characteristics

**Figure 5:**
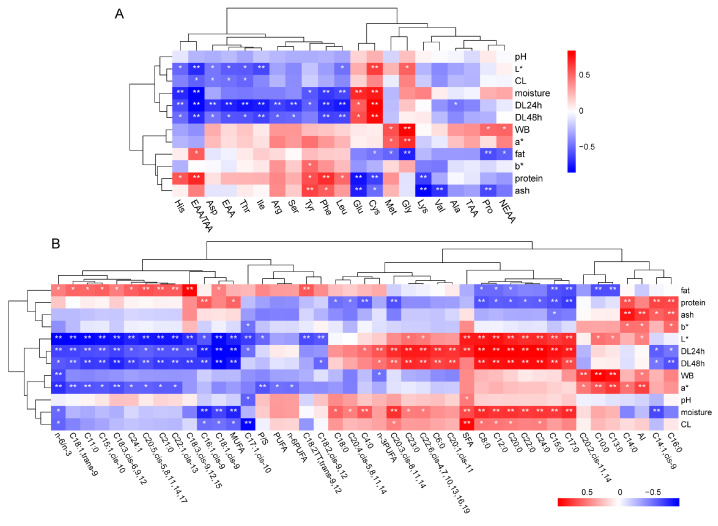
**Correlation between meat quality characteristics.** Pearson correlation analysis was performed to measure the relationship between chemical and physical characteristics and amino acids (**A**) or fatty acids (**B**). Colors in the heatmaps indicate the correlation coefficient, as shown in the scale bar. WB: shear force; DL: drip loss; CL: cooking loss; L*: brightness; a*: redness; b*: yellowness; TAA: total amino acid; EAA: essential amino acid; NEAA: non-essential amino acid; EAA/TAA: essential amino acid/total amino acid; SFA: saturated fatty acid; MUFA: monounsaturated fatty acid; PUFA: polyunsaturated fatty acid; n-6/n-3: n-6PUFA/n-3PUFA; P/S: PUFA/SFA; AI: atherogenic index. Pearson correlation analysis was plotted using R software. *: *p* < 0.05; **: *p* < 0.01.

Correlation analysis was performed to investigate the potential effect of chemical and physical characteristics on the amino acid and fatty acid composition. Our results show that shear force was positively correlated with NEAAs (*p* < 0.05). Protein (*p* < 0.05) and fat (*p* < 0.01) were positively correlated with EAA/TAAs, and fat was negatively correlated with NEAA (*p* < 0.05). Cooking loss (*p* < 0.05), 24 h drip loss (*p* < 0.01), 48 h drip loss (*p* < 0.05), and L* (*p* < 0.05) were negatively correlated with both EAA and EAA/TAA. Interestingly, there is a negative correlation between moisture and EAA/TAA (*p* < 0.01) (Figure 5A).

The analysis between chemical and physical characteristics vs. fatty acid composition showed that SFA is positively correlated with L* (*p* < 0.01), drip loss 24 h (*p* < 0.01), pH (*p* < 0.05), moisture (*p* < 0.01), and cooking loss (*p* < 0.01). In contrast, MUFAs were negatively correlated with L* (*p* < 0.01), drip loss 24 h (*p* < 0.01), drip loss 48 h (*p* < 0.01), moisture (*p* < 0.01), and cooking loss (*p* < 0.05), but positively related with protein (*p* < 0.05). PUFAs were only negatively correlated with a* (*p* < 0.05), and the ratio of PUFAs/SFAs is negatively correlated with a* (*p* < 0.01) and L* (*p* < 0.05). Furthermore, AI was positively correlated with ash (*p* < 0.01), L* (*p* < 0.05), a* (*p* < 0.01), b* (*p* < 0.01), and WB (*p* < 0.05) (Figure 5B).

### 3.6. Principal Component Analysis

**Figure 6:**
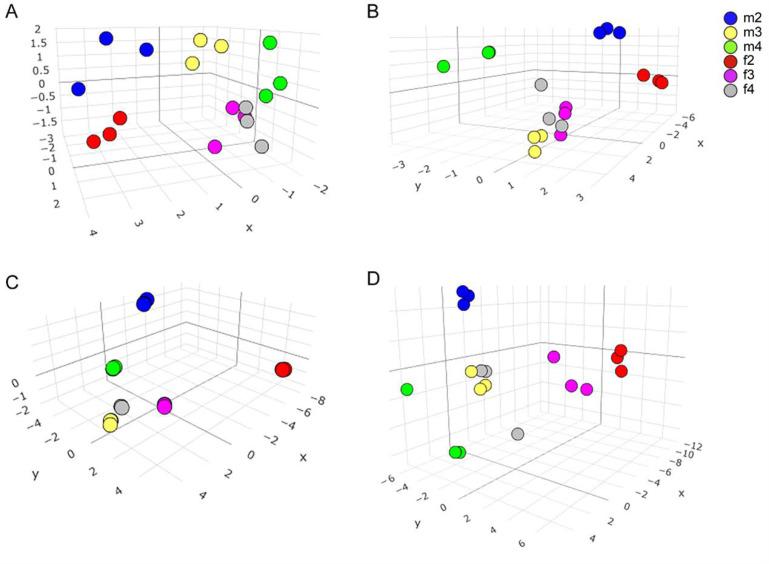
**PCA analysis of characteristics of deer meat.** PCA were performed to analyze the similarities among all the groups by employing data of meat chemical and physical characteristics (**A**), amino acids (**B**), fatty acids (**C**), and all measured parameters (**D**). m2: 2-year-old male deer. m3: 3-year-old male deer; m4: 4-year-old male deer; f2: 2-year-old female deer; f3: 3-year-old female deer; f4: 4-year-old female deer.

Principal component analysis (PCA) was conducted to further investigate the characteristics of Sika deer LD across different age and sex groups. The analysis revealed the distinct separation of 2-year-old male and female deer from the other groups in all analyses (Figure 6A–D), indicating that the younger deer possess unique meat quality characteristics. Notably, when fatty acid data were used for the PCA, data points within the same group were densely clustered (Figure 6C). This suggests that the fatty acid composition of deer meat exhibits minimal variability within each group. Additionally, the clustering of 3-year-old males and 4-year-old females was close, indicating that these two groups are more similar in terms of meat characteristics (Figure 6A–D).

## 4. Discussion

Venison is renowned for its high protein content, low fat, and low cholesterol, contributing to its tender texture and distinctive, fragrant flavor. While scholarly attention has been devoted to exploring the nutritional composition and physicochemical properties of roe deer, red deer, and fallow deer, there remains a notable research gap in characterizing the meat quality of farmed Chinese Sika deer.

The present study systemically investigated the meat quality of Sika deer using LD and showed that age and sex can markedly influence the nutritional profile of Sika deer meat, encompassing parameters such as fat, protein, moisture, amino acids, and fatty acids, as well as physical attributes including meat color, shear force, drip loss (at 24 h and 48 h), pH, and cooking loss.

### 4.1. Chemical Composition and Physical Characteristics

The Sika deer meat is low in fat and high in protein, aligning with the nutritional and health requirements for meat foods [8]. Comparable protein, fat, and ash contents were observed in wild axis deer [13], and fallow deer [24]. Daszkiewicz et al. reported slightly higher protein (22.0–22.4%) and fat (0.56–0.96%) contents in wild red deer from northeastern Poland compared to Sika deer [25]. Meanwhile, the moisture content (74.4–75.2%) and ash content (1.1%) were slightly lower in red deer compared to Sika deer.

Age significantly affects the content of moisture, protein, fat, and ash of venison. As the age of Sika deer increased, there was a gradual decrease in moisture content and a concurrent increase in protein, fat, and ash content. This is probably because, in juvenile deer, the energy absorption primarily contributes to bone development and body growth. In adult deer, where skeleton development is completed, energy absorption is directed towards life-sustaining activities and fat accumulation. Meanwhile, increased body movement results in gradual muscle development. Consistent with these findings, Polak et al. [26] and Żochowska-Kujawska et al. [14] noted that older deer displayed a higher fat content compared to juvenile deer. Additionally, it was found that females aged 13 months to 24 months exhibited the highest protein content compared to females aged 1 month to 12 months [27].

In terms of the effect of sex on the physical traits, the present study showed that 2- and 3-year-old female deer had a higher ash and fat content compared to males, while 4-year-old females had a lower fat content than males. This phenomenon may be attributed to the fecundity status, and the enhanced metabolism of males during the rut, leading to weight loss [28]. Indeed, sex and age have been shown to affect the fat and ash content [25].

Meat quality is intricately linked to various factors, including color, drip loss, tenderness, cooking loss, and pH levels. The color attributes, represented by brightness (L*), redness (a*), and yellowness (b*), significantly influence consumer preferences. Juvenile Sika deer meat, characterized by a higher brightness and redness, aligns well with consumer expectations and suggests potential market attractiveness [29]. Male Sika deer exhibit meat with higher L* and a* values, consistent with the findings in male fallow deer [30]. Overall, deer meat tends to have a redder hue compared to other captive animals such as pigs [31] and sheep [32] because deer are more athletic and have a higher myoglobin content in the muscles [33].

Drip loss, indicative of moisture retention and palatability, varies across species. Sika deer exhibited comparable drip losses to red deer [25], fallow deer (2.86% to 3.12%) [34], African impala (2.9–4.2%) [35], and sheep (1.3–1.5%) [36] compared to the higher drip losses in Sika deer. Our data presented that drip loss gradually decreased with age in both male and female Sika deer, which can be explained by the alterations in muscle fiber density, connective tissue content, and water-binding capacity in older animals. In terms of sex, 2-year-old male Sika deer had a lower drip loss than females, and 4-year-old males had higher losses than females. In contrast, it was reported that drip loss in fallow deer of different feed groups and age groups loss did not differ, which might be caused by the moisture content of the venison [34]. Piaskowska. et al. [24] also indicated that the drip loss of male fallow deer was higher than that of females and that the difference in the drip loss values of 2-year-old Sika deer may be affected by pH or individual differences.

Tenderness, assessed through shear force, significantly influences consumer satisfaction and preference, and the market value of meat [37]. In the present study, the shear force values of Sika deer revealed an intriguing age-related pattern, showing tenderness to be slightly increased with age in females but decreased in males. Previous studies also showed that females have a thinner fiber structure and more tender meat and that older females are also popular with consumers [38]. However, here, we first found that meat tenderness decreased with age in male Sika deer, which differs from reports in fallow deer [39]. This can be explained by the fact that young male Sika deer have insufficient muscle fattening, leading to harder meat. With age advancing, muscles become more tender due to the deposition of intramuscular fat [40].

Cooking losses, correlated with moisture retention, increased with age. Cooking loss was the lowest at 3 years old (26.51% to 39.35%) and gradually increased at 4 years old (41.23% to 42.53%), in line with a report in wild red deer [41]. The cooking loss was similar to that of wild fallow deer (37.2 ± 0.19%) semitendinosus [42] and higher than that of wild red deer (22.17–26.68%) [41]. Cooking loss was higher in male than female Sika deer and cooking losses are known to be related to the meat’s pH and water-holding capacity. Thus, a low pH and high drip loss are usually presented as high cooking loss [43].

pH is one of the most commonly used indices of muscle quality, which impacts the meat’s flavor, color, tenderness, cooking loss, and moisture retention. Low pH often leads to pale, soft exudative meat, and high pH leads to dark, firm, dry meat [44]. The pH of Sika deer ranged from 5.59 to 5.99 with no significant difference in between age, indicating age does not influence the pH of meat in Sika deer.

### 4.2. Amino Acids

Amino acids are compounds that make up the structure of different proteins. The types and amounts of amino acids determine the nutritional value of meat. The present experiment shows that sex and age significantly affected the content of amino acids, especially the essential amino acids. Compared to sex, the effect of age on the content of amino acids is more pronounced. This is probably because the protein content varies more during the growth of the animal. Interestingly, we found that the content of essential amino acids was higher in male than in female Sika deer, contrary to previous findings [13]. Further studies are needed to clarify the differences.

Studies have shown that glutamic acid, alanine, lysine, and aspartic acid are the most abundant amino acids in wild food [15], which is consistent with the present study. Lysine contributes to calcium absorption, which is vital for bone development in children and bone health in older adults; threonine is involved in the synthesis of glycine and serine, which improves the condition of the cardiovascular and immune system; and methionine prevents vitamin B12 deficiency [45]. Compared to the amino acid composition in beef cattle [46], Sika deer have comparable levels of lysine, threonine, valine, leucine, and phenylalanine, and slightly lower levels of methionine, isoleucine, histidine, and arginine. The World Health Organization (WHO) recommends the ratio of essential amino acids/total amino acids at 40% as the ideal protein model [47], and the ratio of essential amino acids/total amino acids in this experiment was 41.35~44.42%, indicating that venison meets the perfect protein model and is rich in nutrients.

### 4.3. Fatty Acids

The composition of fatty acids in meat plays a crucial role in determining its quality, taste, and nutritional characteristics. Major saturated fatty acids in red meat, such as C16:0 and C18:0, significantly impact the proportion of total fatty acids. Atherogenic effects are associated with C14:0 and C16:0, contributing to increased LDL cholesterol synthesis, and total cholesterol levels by inhibiting the expression of LDL receptor genes. In contrast, C18:0 does not have such effects [48]. In the LD muscle of Sika deer, the content of C14:0, C16:0, and C18:0 ranged between 1.59~4.23%, 14.59~28.53%, and 9.63~17.84%, respectively. In comparison, red deer exhibits percentages of 2.23%, 16.19%, and 13.73% [49]. Additionally, the longest loin muscle of female roe deer showcases levels of 1.56%, 24.13%, and 22.05%, while that of red deer ranged from 6.02%, 32.90%, and 17.36%, respectively [12]. This implies that Sika deer meat may confer greater health benefits than red deer meat. Sexual dimorphism is evident, with Polak et al. noting higher levels of C18:0 in females and higher levels of C16:0 in males [26]. Similarly, it was reported that higher levels of total SFAs in females and lower levels of SFAs in 4–5-year-olds compared to 2–3-year-olds [50].

The fatty acid content and composition of meat foods also affect the flavor of meat, with C16:1 and C18:1 being positively correlated with meat flavor [51]. In Sika deer LD, C16:1, cis-9 and C18:1, cis-9 range between 1.54~10.99% and 7.40~15.51%, respectively. In comparison, C16:1 and C18:1 in farmed red deer are 6.9~20.6%, 4.69~13.74% in wild red deer [26], 2.39% and 34.58% in the longest muscle of the loin of female roe deer, and 9.23% and 20.13% in red deer [12], suggesting that the flavor of Sika deer may be slightly lower than that of red deer, and close to that of wild red deer. The C18:1, trans-9 and C18:2TT, trans-9 levels were low in the meat of the Sika deer and were not detected in all 2-year-old Sika deer. Momot et al. reported that the MUFA content of beef was significantly higher in 21-month-old males than in 15-month-olds [52]; Chen et al. also revealed that most of the fatty acids increased progressively with age [53], and Bartoň et al. noted that the MUFA content in steers is significantly higher than that of males [54], which is in agreement with the results of our study. De Smet et al. reported that the fat content affects the fatty acid content of meat, and, as the fat content increases, the various fatty acid contents also increase [55]. In the present study, the fat and most fatty acid contents in female Sika deer were higher than those in males, and the fat content in venison increased gradually with age. Most of the PUFA and MUFA contents were highest in the 3-year-olds of the Sika deer and second highest at 4 years old, which is consistent with the phenomenon described in [55].

C18:2n-6 (LA) and C20:4n-6 (ARA) are the highest PUFAs in Sika deer. A higher LA content generally indicates a better meat flavor [51]. Meanwhile, C20:5n-3 (EPA) and C22:6n-3 (DHA) are difficult to be converted from other fatty acids. Therefore, the intake of EPA and DHA is essential for human health [56]. C18:3n-3 (ALA) is involved in the prevention of cardiovascular diseases, LA lowers LDL two to three times faster than oleic acid [57], and C18:3n-6 (GLA), which cannot be synthesized by the human [58], benefits blood vessels by preventing arterial hypertension [59]. The LA, ARA, EPA, DHA, ALA, and GLA contents were 5.57~24.36%, 3.80~18.11%, 0~0.21%, 0~1.77%, 0~0.51%, and 0~0.14%, respectively, in Sika deer, and 15.12%, 0.34%, 2.90%, 0.50%, 3.40%, and 0.09% in red deer [49]. The LA, ALA, and ARA contents of the longest muscle of the waist of female roe deer were 8.27%, 1.89%, and 2.48%, respectively, while those of red deer were 7.01%, 1.49%, and 21.19%, respectively [12]. In addition, due to differences in diet type, the meat of wild ruminants, including the deer family, contains more PUFAs than that of cattle and sheep [60]. Those data show that Sika deer have a higher content of PUFAs, positioning Sika deer meat as a healthier dietary option.

P/S is an important indicator of meat’s nutritional value. The P/S of female Sika deer of all ages and 3-year-old males are higher than the other groups, indicating a potential to reduce the risk of cardiovascular disease and type 2 diabetes [61]. A healthy diet is suggested to have an n-6/n-3 PUFA ratio of less than 4 [62]. In contrast, the n-6/n-3 ratios of Sika deer are much higher than the standard for a healthy diet and the n-6/n-3 ratios of red deer [12], which is caused by the high n-6 PUFA content and low n-3 PUFA content. Thus, consuming Sika deer meat with foods that are high in n-3 PUFAs is recommended for a balanced diet. It was suggested that an AI index of animal fat between 0.5 and 1.0 is more favorable for human health [63]. The AI indices of female Sika deer at different ages and 3-year-old male Sika deer were within the recommended ranges, and those of 2-year-old male Sika deer were slightly higher than the recommended ranges, which shows that Sika deer meat meets the requirements of a healthy diet.

### 4.4. Correlation and PCA Analysis of Sika Deer Meat Characteristics

Our study reported that cooking loss, drip loss, and L* were negatively correlated with EAAs, EAAs/TAAs, and flavorful fatty acids such as MUFAs, C16:1, and C18:1, indicating that a lower water loss of venison is often associated with a higher nutritional value and better meat texture and flavor. Moisture is negatively correlated with EAAs/TAAs, indicating that, although venison with a higher moisture content might be juicier and more tender, its nutritional value could be compromised. Fat was positively correlated with C18:2TT and C18:3n6, and protein was positively correlated with MUFAs and C16:1, indicating that the conventional nutrient content is positively associated with the functional nutrient content in venison. The positive correlation between C18:3, C18:1, and fat indicate that the flavorful fatty acid content of venison is affected by the fat content, and a higher fat content often leads to improved meat flavor.

Principal component analysis (PCA) is an effective and simple mathematical method in multivariate statistics for addressing high-dimensional complex systems, offering the advantages of comprehensiveness, rationality, and feasibility [64]. PCA has been employed to compare the meat quality in various animals, such as pigs, cows [65], and sheep [66]. Our PCA results in Sika deer meat characteristics revealed that sex and age significantly impact the meat quality and nutrient content of Sika deer, showing marked differences between juvenile deer of different sexes, which corroborates our previous findings.

## 5. Conclusions

The study reported the meat characteristics of farmed Chinese Sika deer meat and highlight the significant influences of age and sex, emphasizing that 3-year-old deer generally exhibit an optimal nutritional value and superior meat quality. Female Sika deer meat is noted for its tenderness, whereas male deer meat typically presents better appearance characteristics. Importantly, Sika deer meat is distinguished by high concentrations of amino acids and proteins, coupled with low levels of fat and trans fatty acids. The ratios of essential to total amino acids and the atherogenic index align favorable with dietary health recommendations. Consequently, the meat of farmed Chinese Sika deer emerges as a valuable source of essential nutritional compounds beneficial for human consumption.

## Figures and Tables

**Table 1:** Chemical characteristics of Sika deer LD and the influence of sex and age.

Parameter	Male (Age)			Female (Age)			*p*-Value		
	2	3	4	2	3	4	Sex	Age	Age*Sex
Moisture (%)	79.36 ± 1.14 ^c^	74.28 ± 1.34 ^ab^	73.59 ± 1.50 ^a^	79.94 ± 1.61 ^c^	77.15 ± 2.44 ^bc^	74.50 ± 0.69 ^ab^	<0.01	0.07	0.41
Protein (%)	20.08 ± 0.71 ^a^	20.59 ± 0.11 ^a^	22.70 ± 0.07 ^b^	19.63 ± 1.06 ^a^	20.51 ± 0.42 ^a^	22.33 ± 0.05 ^b^	<0.01	0.28	0.83
Fat (%)	0.44 ± 0.02 ^a^	1.46 ± 0.02 ^c^	2.19 ± 0.09 ^e^	1.38 ± 0.03 ^b^	2.65 ± 0.02 ^f^	1.64 ± 0.01 ^d^	<0.01	<0.01	<0.01
Ash (%)	1.47 ± 0.02 ^a^	1.44 ± 0.01 ^a^	1.74 ± 0.04 ^c^	1.45 ± 0.01 ^a^	1.53 ± 0.02 ^b^	1.52 ± 0.02 ^b^	<0.01	<0.01	<0.01

Note: Parameters were presented as percentage of fresh matter. A significant *p*-value (*p* < 0.05) suggests that the dependent variable is affected by age, sex, or the interaction between age and sex. Tukey’s method was used as a post hoc comparison to test the differences between each pair of groups. Different superscripts within a row indicate significant differences between groups (*p* < 0.05). *n* = 3 animals per group. Data are shown as mean ± SD.

**Table 2:** Physical characteristics of Sika deer LD and the influence of sex and age.

Parameter	Male (Age)			Female (Age)			*p*-Value		
	2	3	4	2	3	4	Sex	Age	Age*Sex
WB (N)	44.66 ± 9.60 ^b^	21.84 ± 3.58 ^a^	27.95 ± 1.43 ^a^	22.85 ± 6.26 ^a^	24.46 ± 2.78 ^a^	24.75 ± 0.89 ^a^	0.01	<0.01	<0.01
L*	33.04 ± 1.44 ^b^	27.19 ± 0.10 ^a^	31.19 ± 1.71 ^b^	33.17 ± 0.14 ^b^	26.96 ± 0.96 ^a^	27.97 ± 1.02 ^a^	<0.01	0.05	0.04
a*	20.38 ± 5.75 ^c^	10.87 ± 0.40 ^a^	18.28 ± 5.86 ^bc^	13.29 ± 0.69 ^ab^	11.23 ± 1.13 ^a^	10.82 ± 0.45 ^a^	0.04	0.01	0.12
b*	4.21 ± 0.11 ^bc^	2.54 ± 0.93 ^a^	4.91 ± 0.21 ^c^	3.41 ± 0.85 ^ab^	4.25 ± 0.57 ^bc^	3.90 ± 0.40 ^bc^	0.04	0.91	<0.01
pH	5.79 ± 0.14 ^bc^	5.59 ± 0.09 ^a^	5.77 ± 0.05 ^ab^	5.88 ± 0.08 ^bc^	5.84 ± 0.14 ^bc^	5.99 ± 0.12 ^c^	0.05	<0.01	0.38
DL24 h(%)	10.11 ± 0.72 ^b^	7.87 ± 0.52 ^a^	9.27 ± 1.10 ^ab^	12.53 ± 1.07 ^c^	8.50 ± 0.21 ^a^	8.11 ± 0.27 ^a^	<0.01	0.10	<0.01
DL48 h(%)	19.56 ± 0.82 ^c^	18.87 ± 1.26 ^c^	18.60 ± 0.51 ^c^	23.40 ± 0.41 ^d^	16.76 ± 0.48 ^b^	14.86 ± 0.35 ^a^	<0.01	0.07	<0.01
CL (%)	42.25 ± 3.77 ^bc^	26.51 ± 2.26 ^a^	41.23 ± 2.17 ^bc^	44.40 ± 2.40 ^c^	39.35 ± 1.43 ^b^	42.53 ± 2.15 ^bc^	<0.01	<0.01	<0.01

Note: WB: shear force; DL: drip loss; CL: cooking loss; L*: brightness; a*: redness; b*: yellowness. A significant *p*-value (*p* < 0.05) suggests that the dependent variable is affected by age, sex, or the interaction between age and sex. Tukey’s method was used as a post hoc comparison to test the differences between each pair of groups. Different superscripts within a row indicate significant differences between groups (*p* < 0.05). *n* = 3 animals per group. Data are shown as mean ± SD.

**Table 3:** Amino acid profile of the Sika deer LD and the influence of sex and age.

Parameter (%)	Male (Age)			Female (Age)			*p*-Value		
	2	3	4	2	3	4	Sex	Age	Age*Sex
Aspartic acid	7.18 ± 0.07 ^b^	7.26 ± 0.13 ^b^	6.77 ± 0.54 ^b^	6.12 ± 0.10 ^a^	7.01 ± 0.24 ^b^	6.87 ± 0.48 ^b^	0.06	0.02	0.02
Threonine	3.60 ± 0.05 ^b^	3.75 ± 0.06 ^b^	3.51 ± 0.30 ^b^	2.92 ± 0.06 ^a^	3.60 ± 0.12 ^b^	3.50 ± 0.26 ^b^	<0.01	<0.01	0.01
Serine	3.11 ± 0.05 ^b^	3.08 ± 0.06 ^b^	3.02 ± 0.25 ^b^	2.44 ± 0.05 ^a^	3.02 ± 0.10 ^b^	2.90 ± 0.22 ^b^	0.02	<0.01	<0.01
Glutamic acid	12.90 ± 0.17 ^bc^	12.3 ± 0.19 ^b^	10.9 ± 0.88 ^a^	13.89 ± 0.30 ^c^	12.4 ± 0.40 ^b^	11.9 ± 0.92 ^ab^	<0.01	0.02	0.32
Glycine	4.60 ± 0.05 ^c^	3.41 ± 0.06 ^a^	3.68 ± 0.29 ^ab^	3.64 ± 0.07 ^ab^	3.44 ± 0.10 ^a^	3.86 ± 0.24 ^b^	<0.01	<0.01	<0.01
Alanine	5.14 ± 0.04 ^c^	5.02 ± 0.09 ^bc^	4.63 ± 0.40 ^ab^	4.54 ± 0.09 ^a^	4.87 ± 0.17 ^abc^	5.03 ± 0.36 ^bc^	0.66	0.31	0.01
Cysteine	1.15 ± 0.06 ^c^	0.86 ± 0.04 ^b^	0.70 ± 0.07 ^a^	1.84 ± 0.08 ^d^	0.79 ± 0.01 ^ab^	0.89 ± 0.04 ^b^	<0.01	<0.01	<0.01
Valine	4.17 ± 0.07 ^bc^	4.31 ± 0.03 ^c^	3.65 ± 0.33 ^a^	3.91 ± 0.11 ^ab^	4.05 ± 0.10 ^bc^	4.19 ± 0.28 ^bc^	0.10	0.90	<0.01
Methionine	1.45 ± 0.07 ^c^	1.31 ± 0.06 ^b^	1.40 ± 0.12 ^bc^	1.07 ± 0.06 ^a^	1.01 ± 0.03 ^a^	1.32 ± 0.08 ^bc^	<0.01	<0.01	0.01
Isoleucine	3.55 ± 0.04 ^b^	3.64 ± 0.07 ^b^	3.49 ± 0.29 ^b^	3.13 ± 0.04 ^a^	3.65 ± 0.15 ^b^	3.57 ± 0.24 ^b^	0.02	0.19	0.05
Leucine	6.48 ± 0.10 ^b^	6.67 ± 0.17 ^b^	6.73 ± 0.51 ^b^	5.30 ± 0.06 ^a^	6.45 ± 0.30 ^b^	6.44 ± 0.42 ^b^	<0.01	<0.01	0.04
Tyrosine	2.29 ± 0.05 ^b^	2.21 ± 0.11 ^b^	2.55 ± 0.20 ^c^	1.79 ± 0.05 ^a^	2.23 ± 0.14 ^b^	2.15 ± 0.15 ^b^	<0.01	<0.01	<0.01
Phenyl-alanine	3.30 ± 0.02 ^b^	3.35 ± 0.06 ^b^	3.62 ± 0.29 ^c^	2.61 ± 0.02 ^a^	3.20 ± 0.13 ^b^	3.35 ± 0.03 ^b^	<0.01	<0.01	0.01
Lysine	7.34 ± 0.05 ^b^	7.40 ± 0.11 ^b^	6.18 ± 0.50 ^a^	7.32 ± 0.15 ^b^	7.50 ± 0.27 ^b^	7.11 ± 0.46 ^b^	<0.01	0.04	0.04
Histidine	2.26 ± 0.05 ^a^	2.91 ± 0.04 ^c^	2.61 ± 0.20 ^b^	2.27 ± 0.07 ^a^	2.24 ± 0.10 ^a^	2.97 ± 0.19 ^c^	<0.01	0.12	<0.01
Arginine	5.06 ± 0.02 ^b^	4.89 ± 0.08 ^b^	5.13 ± 0.43 ^b^	3.96 ± 0.05 ^a^	4.95 ± 0.21 ^b^	4.72 ± 0.31 ^b^	0.02	0.01	<0.01
Proline	3.28 ± 0.03 ^e^	2.62 ± 0.12 ^c^	1.92 ± 0.15 ^a^	2.27 ± 0.02 ^b^	2.61 ± 0.14 ^c^	2.87 ± 0.18 ^d^	<0.01	0.72	<0.01
TAA	76.85 ± 0.50 ^b^	75.0 ± 1.21 ^ab^	70.4 ± 5.74 ^ab^	69.02 ± 1.18 ^a^	73.0 ± 2.61 ^ab^	73.6 ± 4.69 ^ab^	0.59	0.18	0.04
EAA	32.15 ± 0.10 ^b^	33.3 ± 0.46 ^b^	31.1 ± 2.53 ^b^	28.54 ± 0.48 ^a^	31.70 ± 1.13 ^b^	32.45 ± 1.84 ^b^	0.05	0.07	0.03
NEAA	44.70 ± 0.43 ^b^	41.7 ± 0.76 ^ab^	39.29 ± 3.21 ^a^	40.48 ± 0.70 ^a^	41.36 ± 1.48 ^ab^	41.17 ± 2.85 ^ab^	0.33	0.14	0.04
EAA/ TAA	41.84 ± 0.19 ^b^	44.4 ± 0.12 ^e^	44.2 ± 0.01 ^de^	41.35 ± 0.01 ^a^	43.3 ± 0.05 ^c^	44.0 ± 0.37 ^d^	<0.01	<0.01	<0.01

Note: Parameters were presented as percentage of dry matter. TAA: total amino acid; EAA: essential amino acid; NEAA: non-essential amino acid; EAA/TAA: essential amino acid/total amino acid. A significant *p*-value (*p* < 0.05) suggests that the dependent variable is affected by age, sex, or the interaction between age and sex. Tukey’s method was used as a post hoc comparison to test the differences between each pair of groups. Different superscripts within a row indicate significant differences between groups (*p* < 0.05). *n* = 3 animals per group. Data are shown as mean ± SD.

**Table 4:** Fatty acid profile of Sika deer LD and the influence of sex and age.

Parameter (%)	Male (Age)			Female (Age)			*p*-Value		
	2	3	4	2	3	4	Sex	Age	Age*Sex
C4:0	0.24 ± 0.04 ^b^	0.14 ± 0.01 ^a^	0.13 ± 0.01 ^a^	0.81 ± 0.01 ^d^	0.54 ± 0.36 ^c^	0.11 ± 0.01 ^a^	<0.01	<0.01	<0.01
C6:0	<0.01 ^a^	0.07 ± 0.02 ^b^	0.09 ± 0.01 ^cd^	0.46 ± 0.01 ^e^	0.07 ± 0.01 ^bc^	0.10 ± 0.00 ^d^	<0.01	<0.01	<0.01
C8:0	0.32 ± 0.26 ^b^	0.02 ± 0.01 ^a^	0.10 ± 0.08 ^a^	0.23 ± 0.20 ^b^	0.03 ± 0.01 ^a^	0.02 ± 0.01 ^a^	0.02	0.44	0.77
C10:0	0.50 ± 0.11 ^c^	0.03 ± 0.01 ^a^	0.16 ± 0.04 ^b^	<0.01 ^a^	0.05 ± 0.00 ^a^	0.03 ± 0.00 ^a^	<0.01	<0.01	<0.01
C11:0	<0.01 ^a^	0.01 ± 0.01 ^b^	<0.01 ^a^	<0.01 ^a^	0.02 ± 0.00 ^c^	0.01 ± 0.00 ^b^	<0.01	<0.01	<0.01
C12:0	0.74 ± 0.11 ^c^	0.12 ± 0.01 ^a^	0.31 ± 0.01 ^b^	1.23 ± 0.02 ^d^	0.12 ± 0.01 ^a^	0.14 ± 0.00 ^a^	<0.01	<0.01	<0.01
C13:0	0.41 ± 0.04 ^d^	0.02 ± 0.01 ^ab^	0.14 ± 0.00 ^c^	<0.01 ^a^	0.04 ± 0.00 ^b^	0.03 ± 0.00 ^ab^	<0.01	<0.01	<0.01
C14:0	2.66 ± 0.12 ^e^	2.08 ± 0.10 ^c^	4.23 ± 0.02 ^f^	1.71 ± 0.02 ^b^	1.59 ± 0.01 ^a^	2.31 ± 0.01 ^d^	<0.01	<0.01	<0.01
C14:1,cis-9	0.97 ± 0.03 ^c^	0.97 ± 0 .06 ^c^	1.40 ± 0.02 ^d^	<0.01 ^a^	0.46 ± 0.00 ^b^	0.94 ± 0.00 ^c^	<0.01	<0.01	<0.01
C15:0	0.85 ± 0.04 ^c^	0.56 ± 0.05 ^b^	0.48 ± 0.00 ^a^	1.16 ± 0.01 ^d^	0.47 ± 0.00 ^a^	0.56 ± 0.00 ^b^	<0.01	<0.01	<0.01
C15:1,cis-10	<0.01 ^a^	0.03 ± 0.01 ^b^	<0.01 ^a^	<0.01 ^a^	0.06 ± 0.00 ^d^	0.04 ± 0.00 ^c^	<0.01	<0.01	<0.01
C16:0	26.27 ± 0.75 ^d^	20.67 ± 0.11 ^b^	28.53 ± 0.21 ^e^	14.59 ± 0.14 ^a^	21.44 ± 0.46 ^c^	25.69 ± 0.05 ^d^	<0.01	<0.01	<0.01
C16:1,cis-9	5.14 ± 0.26 ^c^	10.80 ± 0.18 ^e^	7.74 ± 0.04 ^d^	1.54 ± 0.01 ^a^	4.64 ± 0.09 ^b^	10.99 ± 0.04 ^e^	0.49	0.63	0.78
C17:0	1.16 ± 0.47 ^b^	0.56 ± 0.06 ^a^	0.45 ± 0.00 ^a^	1.38 ± 0.01 ^b^	0.50 ± 0.02 ^a^	0.58 ± 0.01 ^a^	<0.01	0.29	0.46
C17:1,cis-10	<0.01 ^a^	0.41 ± 0.03 ^c^	<0.01 ^a^	<0.01 ^a^	0.25 ± 0.01 ^b^	<0.01 ^a^	<0.01	<0.01	<0.01
C18:0	12.06 ± 0.38 ^c^	10.69 ± 0.33 ^b^	9.63 ± 0.06 ^a^	17.74 ± 0.05 ^e^	17.84 ± 0.11 ^e^	13.60 ± 0.05 ^d^	<0.01	<0.01	<0.01
C18:1,trans-9	<0.01 ^a^	0.14 ± 0.01 ^c^	<0.01 ^a^	<0.01 ^a^	0.21 ± 0.01 ^d^	0.12 ± 0.00 ^b^	<0.01	<0.01	<0.01
C18:1,cis-9	10.61 ± 0.61 ^b^	15.41 ± 0.23 ^d^	12.63 ± 0.05 ^c^	7.40 ± 0.05 ^a^	15.21 ± 0.38 ^d^	15.51 ± 0.07 ^d^	<0.01	0.26	<0.01
C18:2TT,trans-9	<0.01 ^a^	0.03 ± 0.00 ^b^	<0.01 ^a^	<0.01 ^a^	0.08 ± 0.01 ^c^	<0.01 ^a^	<0.01	<0.01	<0.01
C18:2,cis-9,12	11.52 ± 0.29 ^d^	10.54 ± 0.27 ^c^	5.57 ± 0.03 ^a^	8.58 ± 0.11 ^b^	24.36 ± 0.29 ^f^	15.02 ± 0.10 ^e^	<0.01	<0.01	<0.01
C18:3,cis-6,9,12	<0.01 ^a^	0.08 ± 0.01 ^b^	<0.01 ^a^	<0.01 ^a^	0.13 ± 0.03 ^c^	0.14 ± 0.01 ^c^	<0.01	<0.01	<0.01
C18:3,cis-9,12,15	<0.01 ^a^	0.18 ± 0.01 ^b^	0.29 ± 0.04 ^c^	<0.01 ^a^	0.51 ± 0.02 ^d^	0.27 ± 0.01 ^c^	<0.01	<0.01	<0.01
C20:0	1.56 ± 0.09 ^d^	0.10 ± 0.01 ^a^	0.49 ± 0.02 ^c^	3.05 ± 0.02 ^e^	0.19 ± 0.02 ^b^	0.14 ± 0.01 ^ab^	<0.01	<0.01	<0.01
C20:1,cis-11	<0.01 ^a^	0.28 ± 0.01 ^c^	0.40 ± 0.03 ^e^	1.41 ± 0.01 ^f^	0.32 ± 0.02 ^d^	0.24 ± 0.01 ^b^	<0.01	<0.01	<0.01
C20:2,cis-11,14	0.63 ± 0.11 ^d^	0.18 ± 0.01 ^b^	0.24 ± 0.01 ^b^	<0.01 ^a^	0.34 ± 0.03 ^c^	0.24 ± 0.01 ^b^	0.04	<0.01	<0.01
C21:0	<0.01 ^a^	0.03 ± 0.00 ^b^	<0.01 ^a^	<0.01 ^a^	0.07 ± 0.01 ^d^	0.04 ± 0.00 ^c^	<0.01	<0.01	<0.01
C20:3,cis-8,11,14	0.97 ± 0.11 ^c^	0.53 ± 0.03 ^a^	0.45 ± 0.03 ^a^	1.94 ± 0.01 ^e^	1.09 ± 0.03 ^d^	0.83 ± 0.04 ^b^	<0.01	<0.01	<0.01
C20:4,cis-5,8,11,14	3.80 ± 0.22 ^b^	6.08 ± 0.13 ^c^	2.23 ± 0.07 ^a^	18.11 ± 0.30 ^f^	12.84 ± 0.28 ^e^	9.39 ± 0.02 ^d^	<0.01	<0.01	<0.01
C22:0	1.52 ± 0.23 ^c^	0.09 ± 0.00 ^a^	0.49 ± 0.01 ^b^	3.41 ± 0.02 ^d^	0.21 ± 0.02 ^a^	0.13 ± 0.01 ^a^	<0.01	<0.01	<0.01
C20:5,cis-5,8,11,14	<0.01 ^a^	0.08 ± 0.00 ^b^	<0.01 ^a^	<0.01 ^a^	0.21 ± 0.02 ^d^	0.11 ± 0.01 ^c^	<0.01	<0.01	<0.01
C22:1,cis-13	<0.01 ^a^	0.07 ± 0.01 ^b^	<0.01 ^a^	<0.01 ^a^	0.16 ± 0.02 ^d^	0.09 ± 0.01 ^c^	<0.01	<0.01	<0.01
C23:0	<0.01 ^a^	0.07 ± 0.01 ^b^	0.35 ± 0.02 ^d^	2.27 ± 0.00 ^e^	0.13 ± 0.01 ^c^	0.10 ± 0.01 ^bc^	<0.01	<0.01	<0.01
C24:0	1.57 ± 0.08 ^d^	0.09 ± 0.00 ^a^	0.51 ± 0.04 ^c^	3.39 ± 0.02 ^e^	0.22 ± 0.04 ^b^	0.14 ± 0.01 ^a^	<0.01	<0.01	<0.01
C24:1	<0.01 ^a^	0.05 ± 0.01 ^b^	<0.01 ^a^	<0.01 ^a^	0.13 ± 0.04 ^c^	0.08 ± 0.01 ^b^	<0.01	<0.01	<0.01
C22:6,cis-4,7,10,13,16,19	<0.01 ^a^	0.07 ± 0.01 ^b^	0.27 ± 0.04 ^d^	1.77 ± 0.02 ^e^	0.18 ± 0.04 ^c^	0.13 ± 0.01 ^c^	<0.01	<0.01	<0.01
SFA	49.74 ± 1.66 ^d^	35.33 ± 0.04 ^a^	46.04 ± 0.29 ^c^	51.55 ± 0.25 ^e^	43.75 ± 0.71 ^b^	43.73 ± 0.14 ^b^	<0.01	<0.01	<0.01
MUFA	16.72 ± 0.89 ^b^	28.14 ± 0.40 ^d^	22.16 ± 0.07 ^c^	10.35 ± 0.06 ^a^	21.44 ± 0.57 ^c^	28.00 ± 0.12 ^d^	<0.01	<0.01	<0.01
PUFA	16.92 ± 0.16 ^b^	17.77 ± 0.36 ^c^	9.06 ± 0.05 ^a^	30.40 ± 0.27 ^e^	39.76 ± 0.14 ^f^	26.13 ± 0.16 ^d^	<0.01	<0.01	<0.01
n-3PUFA	0.00 ± 0.00 ^a^	0.33 ± 0.01 ^b^	0.56 ± 0.07 ^c^	1.77 ± 0.02 ^e^	0.90 ± 0.04 ^d^	0.51 ± 0.02 ^c^	<0.01	<0.01	<0.01
n-6PUFA	16.29 ± 0.07 ^b^	17.23 ± 0.36 ^c^	8.26 ± 0.06 ^a^	28.63 ± 0.26 ^e^	38.44 ± 0.08 ^f^	25.38 ± 0.15 ^d^	<0.01	<0.01	<0.01
n-6/n-3	0.00 ± 0.00 ^a^	52.43 ± 2.99 ^d^	14.81 ± 1.83 ^b^	16.21 ± 0.07 ^b^	42.78 ± 1.62 ^c^	49.98 ± 2.03 ^d^	<0.01	<0.01	<0.01
P/S	0.34 ± 0.01 ^b^	0.50 ± 0.01 ^c^	0.20 ± 0.00 ^a^	0.59 ± 0.00 ^d^	0.91 ± 0.01 ^e^	0.60 ± 0.00 ^d^	0.78	0.76	0.93
AI	1.12 ± 0.01 ^e^	0.63 ± 0.00 ^c^	1.47 ± 0.01 ^f^	0.56 ± 0.00 ^b^	0.46 ± 0.00 ^a^	0.65 ± 0.00 ^d^	<0.01	<0.01	<0.01

Note: Parameters were presented as percentage of dry matter. SFA: saturated fatty acid; MUFA: monounsaturated fatty acid; PUFA: polyunsaturated fatty acid; n-6/n-3: n-6PUFA/n-3PUFA; P/S: PUFA/SFA; AI: atherogenic index. A significant *p*-value (*p* < 0.05) suggests that the dependent variable is affected by age, sex, or the interaction between age and sex. Tukey’s method was used as a post hoc comparison to test the differences between each pair of groups. Different superscripts within a row indicate significant differences between groups (*p* < 0.05). Data are shown as mean ± SD.

## Data Availability

The original contributions presented in the study are included in the article; further inquiries can be directed to the corresponding author.

## Supplementary Materials

The following supporting information can be downloaded at: https://www.mdpi.com/article/10.3390/foods13233978/s1, Table S1: Composition and nutrient levels of the experimental diets (air-dry basis, %).
