# Supplementary material for: Characteristics of Meat from Farmed Sika Deer (Cervus nippon) and the Effects of Age and Sex on Meat Quality
Source: Foods. 2024 Dec 9;13(23):3978. doi: 10.3390/foods13233978 (PMC11640907; doi:10.3390/foods13233978)
Supplement: Supplementary file 1 [file foods-13-03978-s001.zip › foods-3205543-supplementary.pdf]

Supplementary file

Table S1: Composition and nutrient levels of the experimental diets (air-dry basis, %)

Items	Diet
Ingredients	
Extruded corn	26.5
Soybean meal	15.00
Corn germ meal	6.00
Distillers Dried Grains with Solubles	6.00
Alfalfa meal	40.00
Soybean oil	4.50
Sodium bicarbonate	0.10
Salt	0.50
Premix[1)]	1.40
Lys	0.00
Met	0.10
Total	100.00
Nutrient levels[2)]	
ME (MJ/kg)	17.55
DM%	90.03
CP (%)	19.78
EE (%)	4.69
NDF%	51.07
ADF%	14.61
Methionine (%)	0. 60

1): Premix: 1 kg of premix contained the following: MgO, 0.076 g; ZnSO4.H2O, 0.036 g; MnSO4.H2O, 0.043 g; FeSO4.H2O, 0.053 g; NaSeO3, 0.031 g; vitamin A, 2484 IU; vitamin D, 3496.8 IU; vitamin E, 0.828 IU; vitamin K, 0.23 mg; vitamin B1, 0.092 mg; vitamin B2, 0.69 mg; vitamin B12, 0.00138 mg; folic acid, 0.023 mg; nicotinic acid, 1.62 mg; calcium pantothenate, 1.15 mg; CaHPO4, 5.17 g; and CaCO3, 4.57 g. 2): ADF, acid detergent fiber; CP, crude protein; DM, dry matter; EE, ether extract; ME, metabolic energy; NDF, neutral detergent fiber. ME was a calculated value, while the others were measured values.
